# Antiviral nanoparticle ligands identified with datamining and high-throughput virtual screening†

**DOI:** 10.1039/d1ra02293h

**Published:** 2021-07-01

**Authors:** Edward Peter Booker, Ghassan E. Jabbour

**Affiliations:** Department of Electrical Engineering and Computer Science, University of Ottawa Canada gjabbour@uottawa.ca

## Abstract

To help contain the spread of the COVID-19 pandemic and to protect front-line workers, new antiviral measures are required. Antiviral nanoparticles are one such possible measure. Metal nanoparticles made from a variety of metals including gold, silver, and copper can kill or disable viruses that cause significant health problems in humans (such as SARS-CoV-2, HIV, or influenza). To promote interaction between nanoparticles and viruses the stabilizing ligands on the nanoparticle surface should be optimized for docking with proteins. The enormous chemical space of possible nanoparticle ligands makes this optimization experimentally and computationally intractable. Here we present a datamining-based study that searched for nanoparticle ligands that have previously been used, and computationally tested these for their ability to dock with the SARS-CoV-2 spike glycoprotein. These ligands will coat future antiviral nanoparticles to be used outside of the body, not as drugs. The best of these ligands identified were: nitric acid (score: 0.95), phosphoroselenoic acid (score: 0.88), hydroxyammonium (score: 0.83), pyrophosphoric acid (score: 0.81). Inspection of the best of these ligands has suggested design principles for future antiviral nanoparticle ligands, and we suggest further ligands based on these principles. These results will be used to inspire further *in vitro* and *in silico* experimentation to accelerate the development of antiviral nanoparticles.

## Abstract 2

To help contain the spread of the COVID-19 pandemic and to protect front-line workers, new antiviral measures are required.

## Graphical abstract

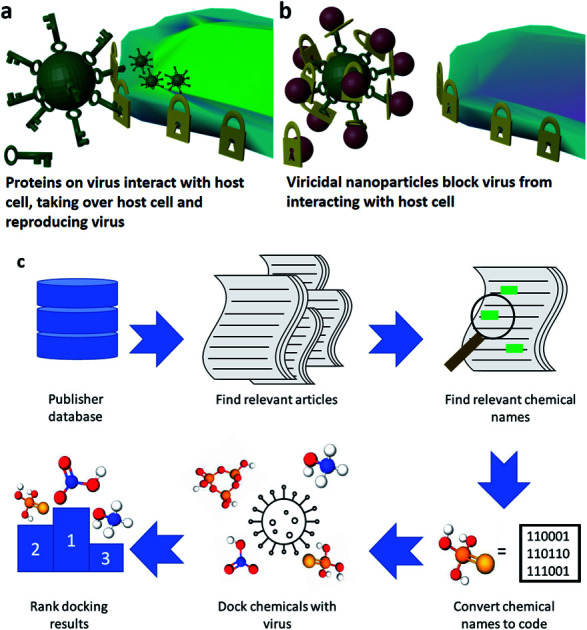

## Introduction

**Fig. 1:**
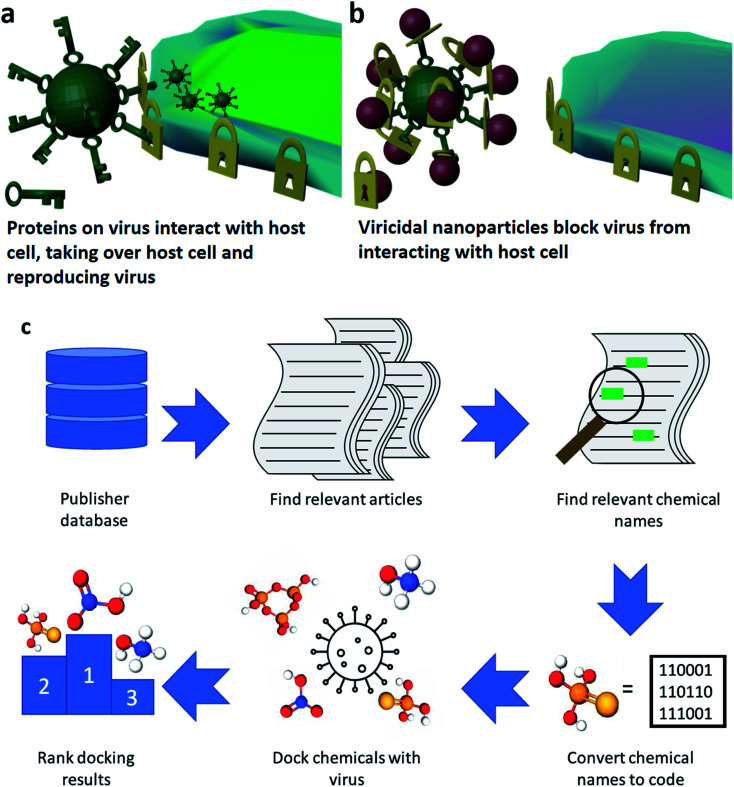
(a) Cartoon of how the coronavirus interacts with host cells reproducing the virus. The spike glycoprotein (keys) of the virus interacts with the ACE2 enzymes (locks) to gain access to the cell (b) cartoon of antiviral nanoparticles using their ligands (locks) to stop the virus from accessing the cell. (c) Illustration of the datamining pipeline to discover novel nanoparticle ligands for use in antiviral nanoparticles. The database of a scientific publisher is searched for articles pertaining to our topic, nanoparticles. These articles are saved in a computer-readable format and consequently mined for relevant chemical names (relating to ligands). These chemical names are computationally docked with the spike glycoprotein on the SARS-CoV-2 virus. These docking poses are compared and ranked to find the best candidate nanoparticle ligands.

The ongoing coronavirus pandemic and the relatively low effectiveness of traditional approaches to dealing with it underscore the need for novel approaches to contain and kill viruses. In medicine, prevention is preferable to a cure and viricidal materials are among the best ways to prevent people catching viruses in the first place. Viricides operate by killing viruses on surfaces through coatings and disinfectants as well as preventing their airborne transmission through active face masks.^1,2^ To help slow the spread of the SARS-CoV-2 virus and other viruses, broad-spectrum viricides that can be easily adapted are needed. SARS-CoV-2, the cause of the COVID-19 illness, and other corona viruses are coated with spike glycoproteins. These proteins bind to the ACE2 enzyme coating smooth muscle cells or alveolar cells like a key fitting a lock (Fig. 1a).^3^

Over the last 15 years, metal and metal-oxide nanoparticles (NPs) have emerged as a novel class of highly customizable broad-spectrum viricide. These NPs have been shown to deactivate a wide range of viruses that present significant public health threats, including HIV,^4^ herpes,^5^ influenza,^6^ and arenavirus.^7^ The model for how NP viricides deactivate viruses is by binding to the external glycoproteins, which prevents the viruses being able to dock with any cells so they cannot infect cells or reproduce (Fig. 1b).^8^ The mechanisms driving NP deactivation of viruses rely on different aspects of these NPs. These aspects include the constituent elements of the NP (Zn, Au, Ag, or Cu^6,9–11^), their size (three to fifty nanometers^12,13^), and coatings (coated with shells, ligands or bare NPs^5,13,14^). To stabilize nanoparticles in solution, they often need to be coated in a chemical that allows them to be stable in a solvent without the nanoparticles dissolving or agglomerating together.^15^ These can be referred to as surfactants, coordinating solvents, or ligands. Improving the performance of a material by varying just one of these properties (size, composition, coating, *etc.*) presents a significant challenge. With three or more properties to vary there are hundreds of thousands of combinations of materials of which many will give some antiviral effect. Screening these to find a scalable and effective viricidal solution will require down-selecting candidate materials for the ideal formulation for commercialization.

High-throughput and automated experiments can accelerate materials discovery.^16*a*,*b*^ However, in the context of devices, biomaterials applications or finding new reagents, experiments can be time-consuming, expensive, or have bottlenecks due to procurement that prevent screening many tens or hundreds of candidate materials. An active area of research is the application of machine learning and datamining to pre-select experiments or reagents to facilitate rapid prototyping and development of new materials and compounds.^17^

In the context of nanomaterials, there are numerous published papers and different methods of synthesis that it is impractical for researchers to read all the literature. The Royal Society of Chemistry alone has published over 118 000 articles or chapters that respond to a search for nanoparticles.^18^ A recently developed text classification tool (ChemDataExtractor^19^) lets researchers pass whole articles to computer software that can identify chemical names. Fortuitously, the RSC's bibliography is available electronically upon request. In this paper, we used the RSC's electronic archive and the CDE tool to search through several thousand of the most recent research papers on nanoparticles. We identified several tens of thousands of compounds that could be candidates as nanoparticle ligands, and we used protein docking simulations to find out which of these ligands is best docked with the SARS-CoV-2 virus spike glycoprotein.

Our approach is based on how well identified nanoparticle ligands dock with the spike protein, and not on their suitability as ligands while docking with the protein, nor their specificity as components in drugs. It may be that some of the identified species may destroy the target nanoparticles, or be unsuitable for toxicity reasons. However, the results here still inspire other potential ligands. Further, the methods used here do not select for docking with other SARS-CoV-2 proteins or other interactions of nanoparticles and viruses. The goal is to provide a first pass selection screening to choose nanoparticle ligands that may augment any other mechanisms by which nanoparticles deactivate viruses by allowing them to bind more effectively.

The design rules suggested by this study should provide generic advice for the synthesis of antiviral nanoparticles: a large number of functional groups that can form hydrogen bonds with virus proteins to prevent those proteins from bonding with host cells. These conclusions are very speculative, however, and to validate them extensive *in vitro* and *in silico* experimentation is required which goes beyond the scope of this work. Further, this work highlights that data-mining-based high-throughput screening may provide valuable insight to accelerate experimental programs.

## Methods

This study was inspired by the recent high-throughput drug design project carried out by the Galaxy project.^20^ The Galaxy project is an open-source scientific computing tool that provides easy access to bio- and chemoinformatics resources.^21^ This large collaborative effort mission is to identify compounds that may effectively dock with the SARS-CoV-2 virus. This project combined highly detailed crystallographic experiments with the structures of several tens of thousands of chemicals that may dock with the main protease of the SARS-CoV-2 virus. Using the rDock software package,^22^ they predicted the best docking poses between their molecules and the protein in question, and ranked the results to provide advice for future viral treatments. The rDock package generates minimum energy poses for ligands interacting with proteins.

The ChemDataExtractor (CDE) package was developed by the Cole group at the University of Cambridge.^23^ This package allows for the analysis of text documents to extract the chemicals that are mentioned. In this study we used the selenium python package to search the RSC publishing website for article corresponding to the term nanoparticles. These articles were read as HTML files by the CDE package which looked for paragraphs containing the terms ligand, surfactant or coordinating solvent, as these were considered to be sufficiently broad to capture all the necessary chemicals which stabilized the nanoparticles in the articles. These paragraphs were then searched for chemicals. The found chemical names were filtered to remove any lone elements, and the rest were converted first to CID numbers, then to SMILES structures. As in the Galaxy project study, we varied the charge states of these SMILES structures from a pH of 4.4 to 10.4. This provided a list of around 12 000 unique chemicals (see ESI†). We then determined the 3D structures of these molecules using the rdkit tool. This process is illustrated in Fig. 1c, and a detailed breakdown of the methodology is available in the ESI.†

Our study used similar principles, and made use of many of the same tools. We took the structure of the spike glycoprotein, which has been suggested from published studies as the part of the coronavirus that initially binds to the host.^24^ The part of the host that the virus appears to bind to is the ACE2 layer. This is a large protein, and many parts of it interact with the spike glycoprotein. To simplify the analysis, the largest fragment of the ACE2 layer that interacts with the spike glycoprotein was chosen and this was used to identify the active site in the glycoprotein (from residue 21 to 61 of the ACE2 layer as informed by Benton *et al.*,^25*a*^ see ESI document 2† for the ACE2 fragment used, and ESI document 4† for the ACE2 fragment used). This active site was used in our rDock analyses. The ∼12 000 chemicals were converted into the SDF format, suitable for the rDock package, and then the docking was carried out. As in the initial Galaxy project study we then used the xchem rdsort tool, which is a machine learning-based method,^25*b*^ to score the docked poses of our identified nanoparticle ligands. This provided a ranked list of the nanoparticle ligands that were assessed manually.

## Results

The datamining procedure that we carried out was able to read around 18 000 academic papers and scan them for chemical names in the same paragraph as the words: ‘ligand’, ‘surfactant’, or ‘coordinating solvent’. The identified chemicals were filtered, converted to CIDS numbers, then SMILES, followed by construction of 3-dimensional chemical structures. The majority of the resultant chemicals found were real chemicals, however there were names, addresses and similar words that were picked up as chemicals. However, most of these fake chemicals were filtered out by the various conversion steps, while any that somehow made it through the filters would have contributed to a marginal additional computational load. Once these various conversions had taken place, around 11 393 chemicals were able to be successfully docked with the identified active site of the spike glycoprotein. The lists of chemical names, SMILES, and ranked chemicals are given as ESI,† and can be found in the GitHub repository linked to this paper. We propose to use these datasets to establish generative models to attempt to design new ligands which are able to dock with viruses in the future.

**Fig. 2:**
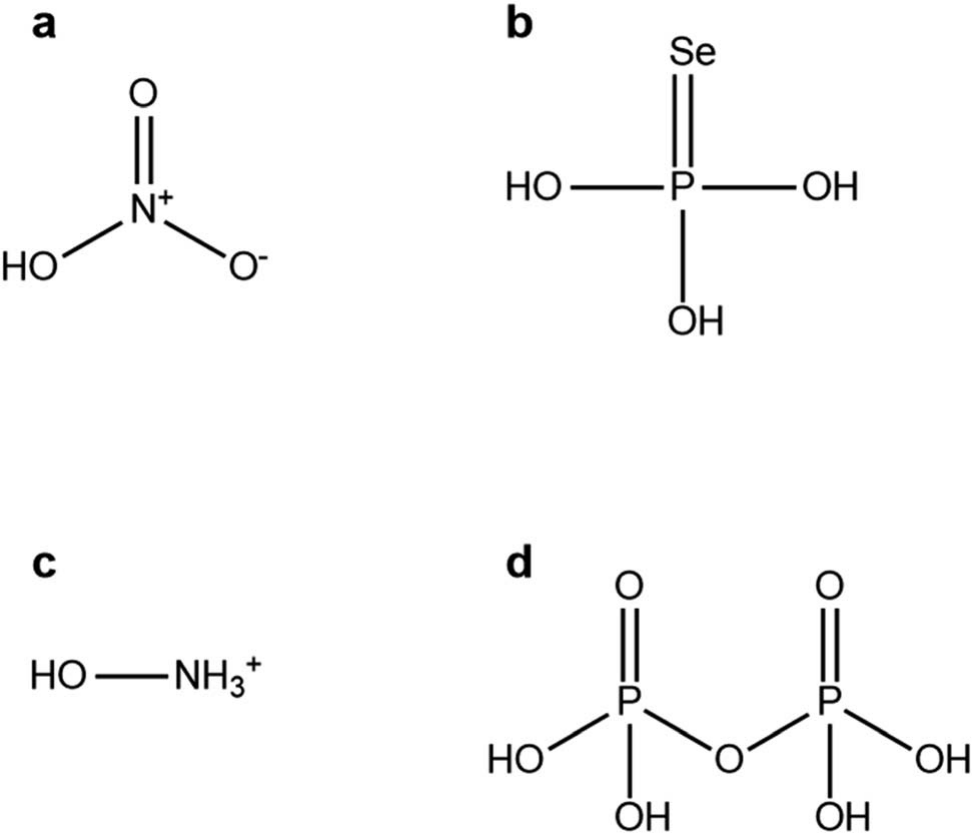
Molecular structure drawings of the four best small molecules and ions found in the datamining study to identify antiviral nanoparticle ligands. (a) nitric acid (score: 0.95) (b) phosphoroselenoic acid (score: 0.88) (c) hydroxyammonium (score: 0.83) (d) pyrophosphoric acid (score: 0.81).

The results from the rDock procedure gave a ranked list of the chemicals which may be used as nanoparticle ligands. Molecules based on the acids of nitrogen and phosphorus were the best small molecules (less than 20 non-hydrogen atoms). Fig. 2 presents the top four of these small molecules.

What can be seen from these molecules is that large numbers of very polar bonds should provide good docking opportunities with the amino acids in the spike glycoprotein. While this is not a particularly surprising result, it is an interesting one. Very polar acid and amine groups will also allow these molecules to both form many different bonds with the surface of ionic nanoparticles (*e.g.*, PbSe^26*a*^), so there will be potentially many different groups of each ligand exposed to the spike protein.

**Fig. 3:**
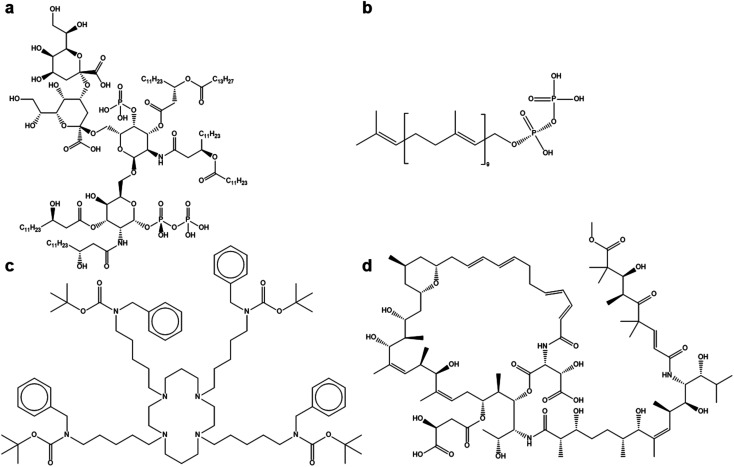
The four best larger ligand molecules found in the datamining study to identify antiviral nanoparticle ligands. (a) (Kdo)2-lipid A 1-diphosphate (score: 0.99) (b) decaprenyl diphosphate (score: 0.98) (c) tetra-*tert*-butyl(1,4,8,11-tetraazacyclotetradecane-1,4,8,11-tetrayltetrapentane-5,1-diyl)tetrakis(benzylcarbamate) (score: 0.96) (d) 4-({15-[carboxy(hydroxy)methyl]-24,28,30-trihydroxy-18-[2-hydroxy-1-({3,7,11,13-tetrahydroxy-12-[(7-hydroxy-9-methoxy-4,4,6,8,8-pentamethyl-5,9-dioxo-2-nonenoyl)amino]-2,6,8,10,14-pentamethyl-8-pentadecenoyl}amino)propyl]-19,23,25,27,29,34-hexamethyl-13,16-dioxo-17,36-dioxa-14-azabicyclo[30.3.1]hexatriaconta-3,5,9,11,22,26-hexaen-20-yl}oxy)-2-hydroxy-4-oxobutanoic acid (score: 0.95).

In addition to small molecules, some of the best larger ligands identified in this study also present interesting options for antiviral nanoparticle ligands. The most interesting examples of these are shown in Fig. 3:

Again, these sizable ligands indicate that large numbers of groups enabling polar and hydrogen bonding between the ligand and the spike glycoprotein. What can also be seen is that these large ligands do not have fully conjugated backbones, which may allow the ligands to sufficiently rotate and distort to give good docking postures with the virus protein. These molecules may be less ideal for the development of antiviral solutions on a large scale, but they do inspire further investigation of large molecules that may bind well with the virus. It should be noted the overall dipole moment of the molecule or ion is not as important as the functional groups themselves. For example, decaprenyl diphosphate scores higher than pyrophosphoric acid (0.98 compared to 0.81) but clearly the long aliphatic end group on decaprenyl diphosphate renders the total molecule less polar than pyrophosphoric acid.

**Fig. 4:**
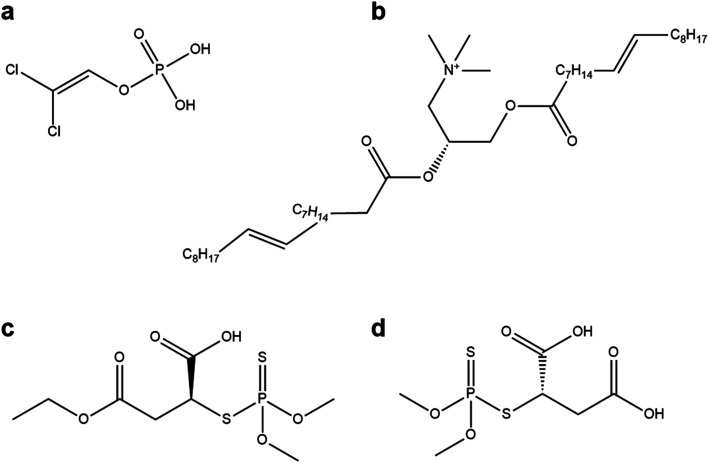
Molecular diagrams and associated scores of the molecules inspired by the results from the datamining campaign. (a) 2,2-Dichlorovinyl dihydrogen phosphate (score: 0.21) (b) Hamburg esterquat (score: 0.21) (c) malathion α-monoacid (score: 0.13) (d) malathion diacid (score: 0.09).

We elaborated on our findings that molecules with large aliphatic groups and many polar groups tended to dock best with the spike glycoprotein to explore the potential for other molecules to act as ligands for antiviral nanoparticles. Five groups of molecules were investigated, related to: famotidine, sucrose, remdesivir, fabric softeners and trimetaphosphoric acid. Famotidine is an antacid that is purported to have significant benefits against COVID-19 (ref. 16*b*) and several structures related to famotidine were investigated, and indeed have been suggested from other *in silico* studies as good molecules to dock with different SARS-CoV-2 proteins (3CLpro,^26*c*^ and the papain-like protease^26*d*^). Sucrose and related sugars have been used to coat nanoparticles^27^ and are very widely available, so sucrose and sucrose esters were also investigated. The drug remdesivir has been used as COVID-19 treatments and may inhibit the virus.^28^ By inspection of the structures of the best ligands identified in the previous sections, it was hypothesized that the surfactant chemicals in fabric softeners (which prevent triboelectric charging in tumble dryers)^29,30^ may be interesting candidates. Trimetaphosphoric acid, a widely available surfactant, was one of the high-performing data-mined candidates, so we also investigated compounds related to this structure. The docking results of these compounds and their related groups can be seen in Fig. 4, along with diagrams of these molecules. The full list of compounds investigated and their scores may be seen in the ESI.†

The results from these inspired molecules suggest that, although many polar functional groups are necessary for successful docking with virus proteins, this alone is not sufficient, and molecules like remdesivir (Fig. 4c) and famotidine (Fig. 4d) do not perform as effectively as other molecules identified in this survey, they both scored less than 0.01. We stress that while our results suggest that these molecules will dock poorly with the spike glycoprotein as we have investigated it, they do not account for other protein bindings, chemical reactions or other pharmacological effects of these compounds. Our findings for the compounds related to 2,2-dichlorovinyl dihydrogen phosphate and 1,2-dioleoyl-3-trimethylammonium propane are also encouraging, and prompt the investigation of additional ligands related to these structures.

## Discussion

The datamining study carried out has provided several candidate molecules to use as nanoparticle ligands. The most appropriate of these are: nitric acid (2a), phosphoroselenoic acid (2b), pyrophosphoric acid (2d), decaprenyl diphosphate (3b) and Hamburg esterquat (4b). In the case of compounds 2a and 2d, they were likely used as reagents for nanoparticle synthesis, but merely in the same paragraph as the word surfactant or ligands.

Higher ranked docking compounds (4a, 4c and 4d) have been excluded as candidates based on the impracticality of using them as nanoparticle ligands, either due to high cost (4a), or the fact that they cannot be sourced commercially at this time, and so would be unsuitable for rapid scaling (4c, 4d).

The main drawback with our approach is that it selects molecules based solely on how well they dock with the spike protein, and not on their suitability as ligands. In certain conditions, attempting to use these very strong oxidizers as ligands may result in the destruction of the target nanoparticles. The results here inspire other potential ligands. Small organic molecules with nitric acid groups, such as 5-thio-2-nitrobenzoic acid (used in nanoparticle synthesis by Lai *et al.*^30^) may be suitable. The same procedure used in this study suggested this molecule would dock with the spike protein with a score of 0.078. The trimetaphosphate ion may be a suitable nanoparticle ligand that would interact well with the spike protein, but not destroy the nanoparticles in the process. Sodium trimetaphosphate is used in the food industry, and so may be suitable for incorporation in breathing apparatus, for example.

These suggested ligands shall be used with the leading antiviral metallic nanoparticle cores to produce fast and highly effective antiviral solutions, similar to work carried out by our group that is currently in press. The datamining aspect of this study returned chemicals that were in the same paragraph as the word ligand. This was done in the hope that the chemicals had been used as nanoparticle ligands before. The full list of chemicals found and the associated DOI references are found in ESI document 1.† The scores, and docked ligand-amino acid poses of the candidate ligands have been included in ESI documents 4 and 5 respectively.† Many of these molecules have been used in nanoparticle syntheses before, and as such the incorporation into our fabrication methods should be relatively straightforward. Further, as the small molecules identified are often used in industrial applications (nitrobenzoic acid, in the synthesis of procaine,^31^ sodium trimetaphosphate, as an additive to chewing gum^32^) they present cost-effective molecules that will facilitate the scale-up of production of antiviral nanoparticles for efforts to protect front-line workers and the general public in the current and future pandemics. The results of our study may be compared to the study by Mulholland and coworkers.^31*b*^ Molecules such as vitamin K and dexamethasone were successful with their method. These have similar features to the structures identified in our study: highly polar functional groups and aliphatic tails (in the case of vitamin K), which support the methodology pursued here.

## Conclusions

These results also suggest design rules for antiviral nanoparticle ligands in general. Ligands should be able to conform to the virus proteins, be small, and provide many opportunities for forming bonds with the virus proteins. These opportunities can come from amine, carboxylic acid, phosphorous acid, thiol and nitric acid groups, amongst others. In addition, these end groups allow the ligands to bind to the surface of the antiviral nanoparticle, thus reducing the toxicity of the ligands themselves, which in any case should be present at relatively low molar concentrations.

Based on from these results, we will synthesize several batches of antiviral nanoparticles, both with silver cores and with silica cores, to determine whether it is the reduction of the virus proteins by the nanoparticle core or the binding of the protein to the reactive ligands that causes these nanoparticles to deactivate viruses so effectively. These results will be complemented by additional molecules to provide a training set for the development of a generative adversarial network to design and evaluate potential new ligands that could be synthesized or purchased for next generation antiviral nanoparticles.

Using a basic data mining approach, we have identified guiding principles for the design of antiviral nanoparticle ligands that will be used to help determine the mechanisms of action for antiviral nanoparticles and to improve their ability to kill viruses. We have also seen that our method of discovering and testing chemicals to be used for nanoparticle ligands has been dramatically accelerated by electronic access to scientific publications. We also suggest that all publishers should provide means to carry out datamining campaigns for their research projects, and that experimentalists should use these tools to accelerate their literature reviews and lower the cost of their research.

## Conflicts of interest

There are no conflicts to declare.

## Supplementary Material

RA-011-D1RA02293H-s001

RA-011-D1RA02293H-s002

RA-011-D1RA02293H-s003

RA-011-D1RA02293H-s004

RA-011-D1RA02293H-s005

RA-011-D1RA02293H-s006
