# Supplementary material for: Antiviral nanoparticle ligands identified with datamining and high-throughput virtual screening
Source: RSC Adv. 2021 Jul 1;11(37):23136–43. doi: 10.1039/d1ra02293h (PMC9034338; doi:10.1039/d1ra02293h)
Supplement: RA-011-D1RA02293H-s006 [file RA-011-D1RA02293H-s006.pdf]

Electronic Supplementary Material (ESI) for RSC Advances.
This journal is © The Royal Society of Chemistry 2021

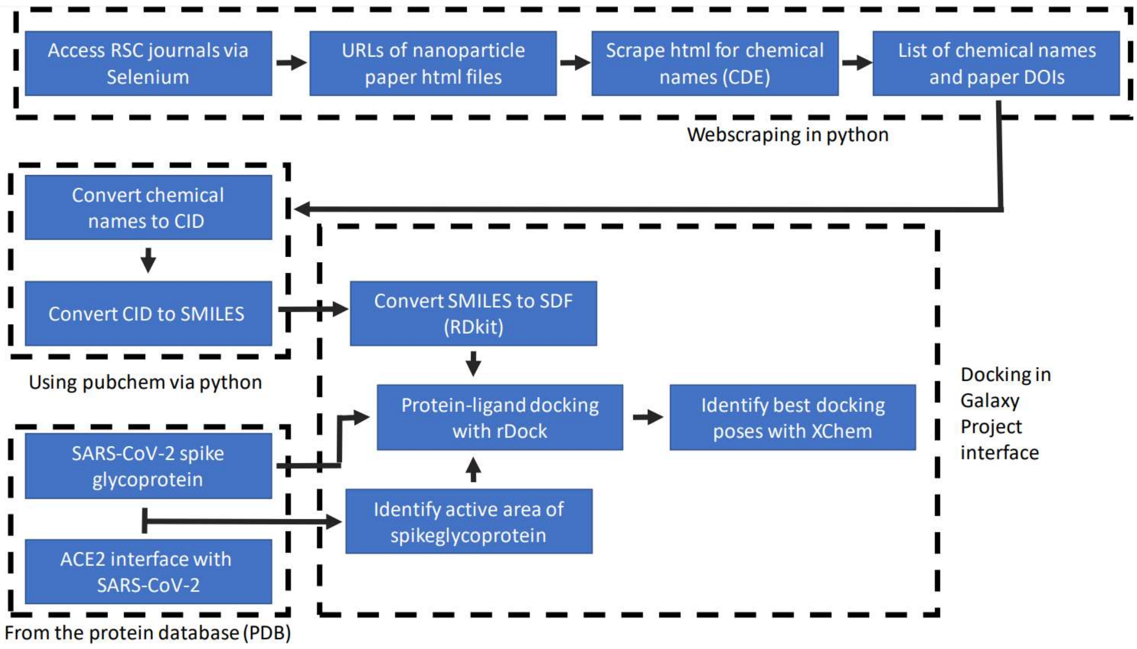

Supplementary figure: detailed pipeline of how data mining was carried out in this study.

The code for the python sections of this pipeline can be found at the relevant github repository: https://github.com/epb378/Datamining_for_ligands

The selenium headless browser library was used to mine for articles from RSC Publishing's web interface, with the permission of the RSC Publishing. These articles were downloaded as html files, and the CDE library was used to scrape these articles for chemicals that appeared in the same paragraphs as the keywords: ligand, surfactant, or coordinating solvent. The scraper then returned a list of chemical names found and their metadata (doi, author names, date, journal, etc.). This list of chemicals and metadata was condensed into a list of unique chemicals and the dois for the articles that each of these chemicals appeared in. The pubchem website was queried to find CIDs from chemical names, and then SMILES structures from those CIDs. Each chemical name may have returned multiple CIDs, so only the first was kept. The SMILES were exported in a .smi format for use in the Galaxy platform. The structure of the ACE2 interface as it coordinated with the SARS-CoV-2 spike protein and the spike protein structures were taken from the protein database. These structures were exported into the Galaxy platform. rDock simulations were carried out in the Galaxy platform with the list of SMILES we had found and these protein structures. These dockings were ranked with the XChem tool on the Galaxy platform.
